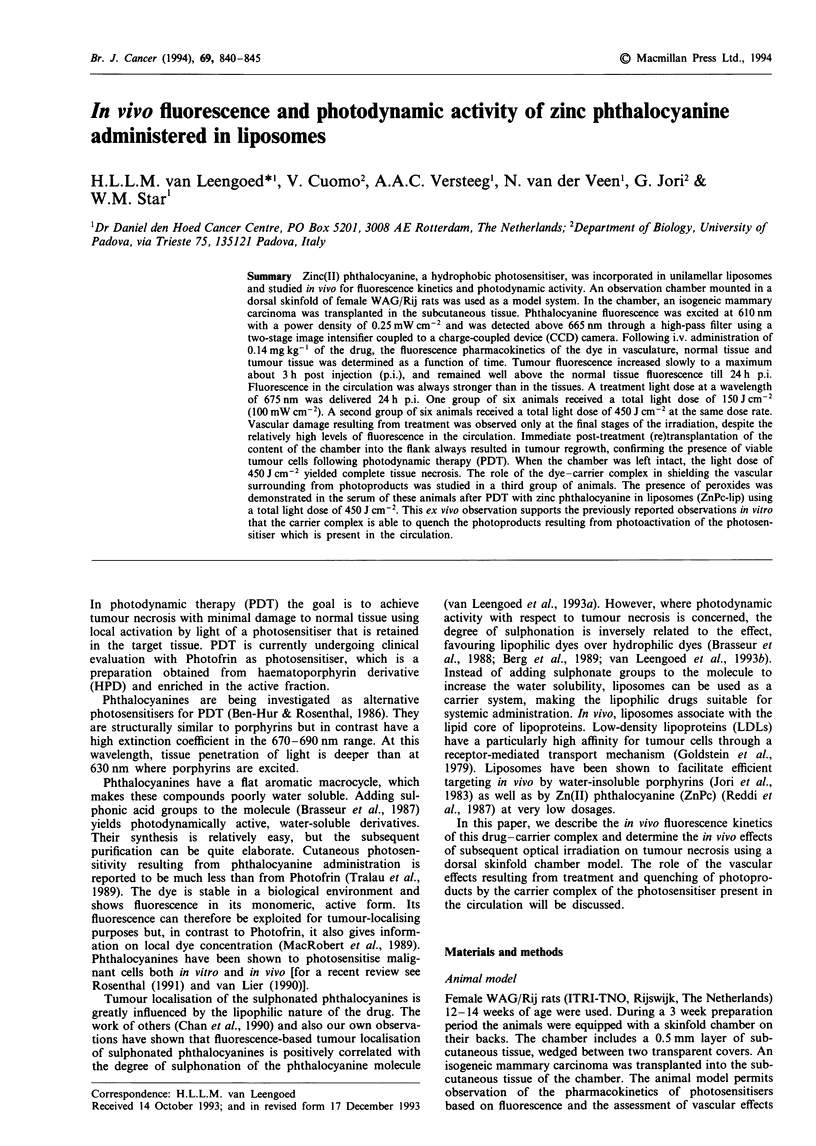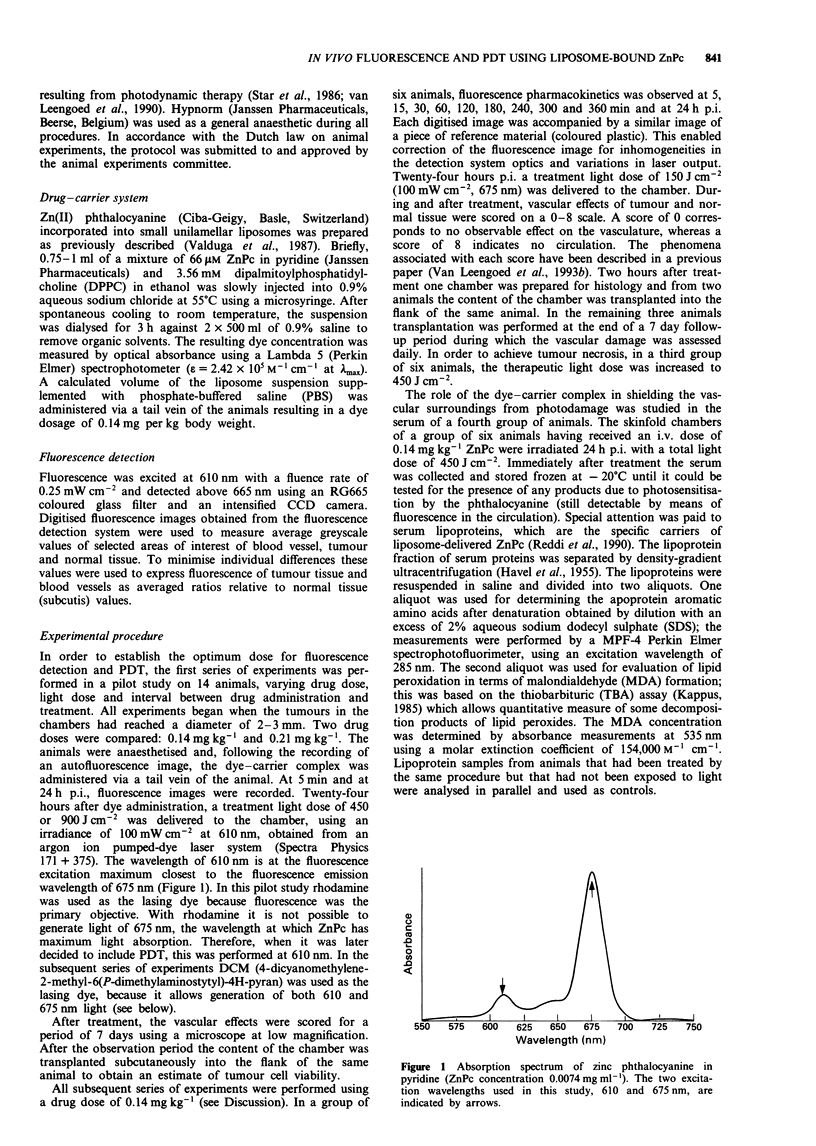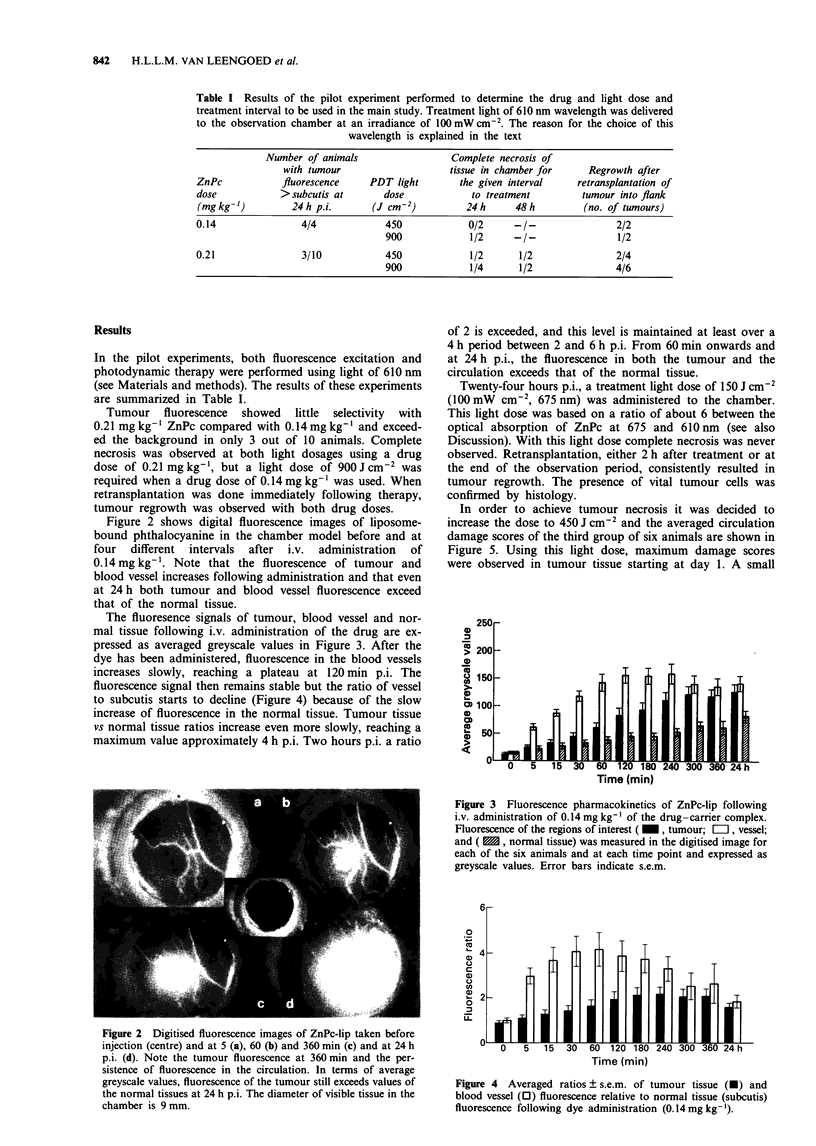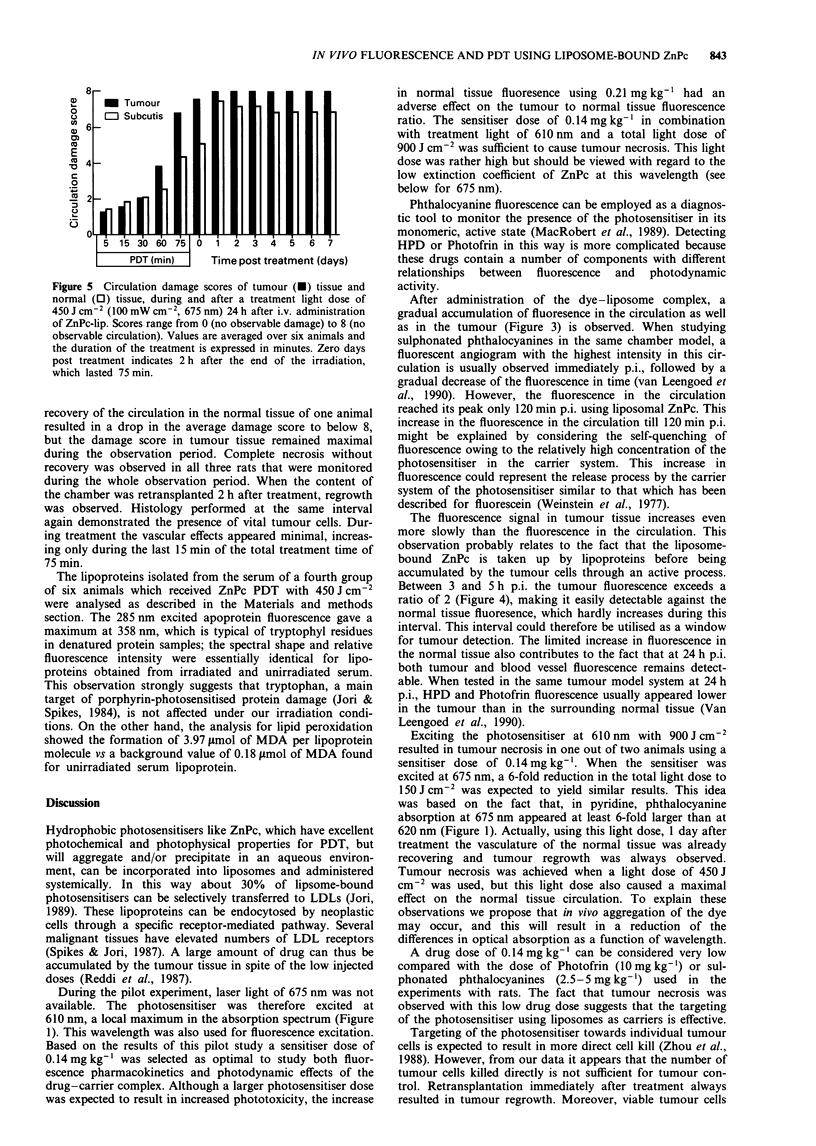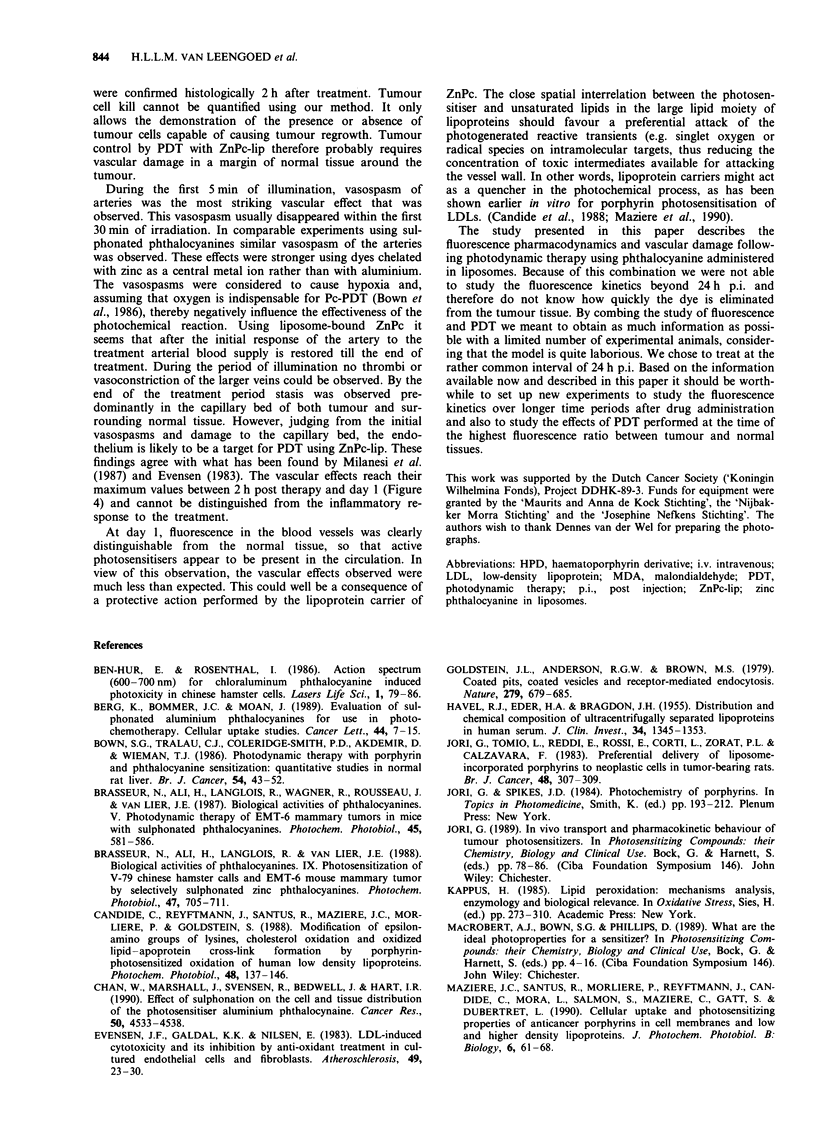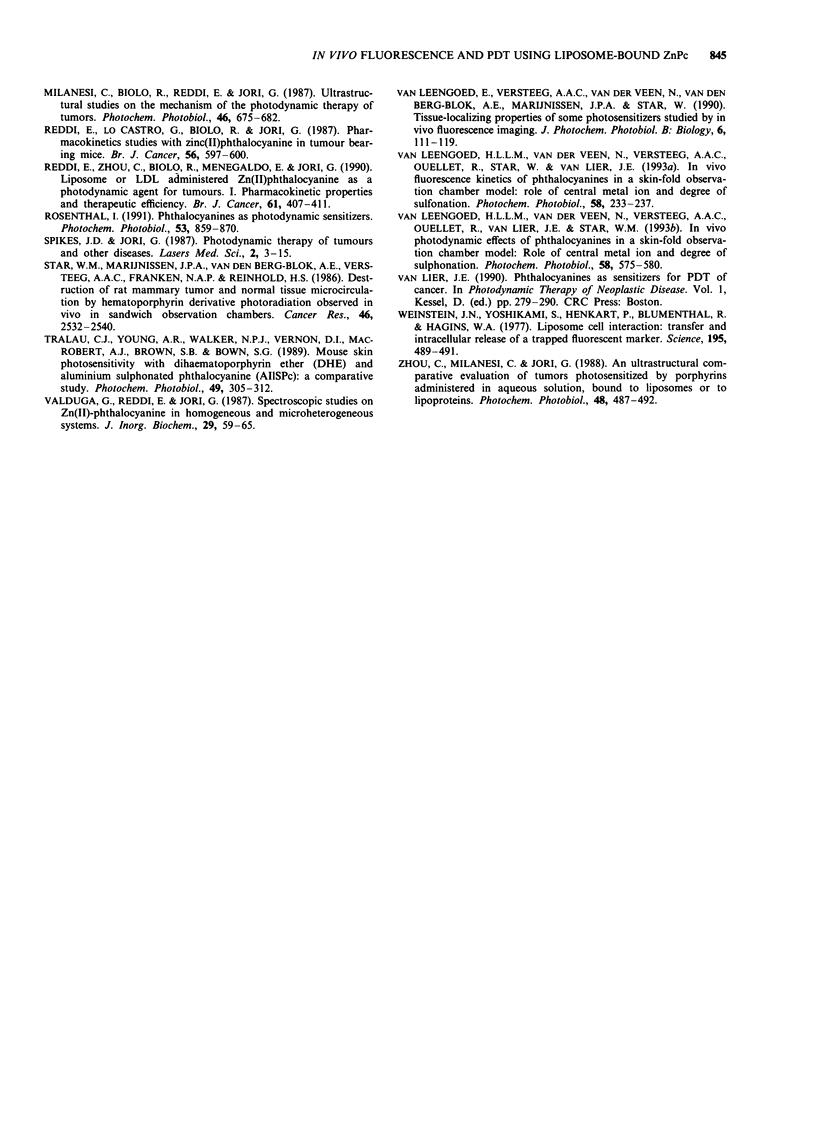# In vivo fluorescence and photodynamic activity of zinc phthalocyanine administered in liposomes.

**DOI:** 10.1038/bjc.1994.163

**Published:** 1994-05

**Authors:** H. L. van Leengoed, V. Cuomo, A. A. Versteeg, N. van der Veen, G. Jori, W. M. Star

**Affiliations:** Dr Daniel den Hoed Cancer Centre, Rotterdam, The Netherlands.

## Abstract

**Images:**


Br. J. Cancer (1994), 69, 840–845 © Macmillan Press Ltd., 1994

### *In vivo* fluorescence and photodynamic activity of zinc phthalocyanine administered in liposomes

H.L.L.M. van Leengoed*[1], V. Cuomo[2], A.A.C. Versteeg[1], N. van der Veen[1], G. Jori[2] & W.M. Star[1]

[1]*Dr Daniel den Hoed Cancer Centre, PO Box 5201, 3008 AE Rotterdam, The Netherlands;* [2]*Department of Biology, University of Padova, via Trieste 75, 135121 Padova, Italy*

**Summary** Zinc(II) phthalocyanine, a hydrophobic photosensitiser, was incorporated in unilamellar liposomes and studied *in vivo* for fluorescence kinetics and photodynamic activity. An observation chamber mounted in a dorsal skinfold of female WAG/Rij rats was used as a model system. In the chamber, an isogeneic mammary carcinoma was transplanted in the subcutaneous tissue. Phthalocyanine fluorescence was excited at 610 nm with a power density of 0.25 mW $cm^{-2}$ and was detected above 665 nm through a high-pass filter using a two-stage image intensifier coupled to a charge-coupled device (CCD) camera. Following i.v. administration of 0.14 mg $kg^{-1}$ of the drug, the fluorescence pharmacokinetics of the dye in vasculature, normal tissue and tumour tissue was determined as a function of time. Tumour fluorescence increased slowly to a maximum about 3 h post injection (p.i.), and remained well above the normal tissue fluorescence till 24 h p.i. Fluorescence in the circulation was always stronger than in the tissues. A treatment light dose at a wavelength of 675 nm was delivered 24 h p.i. One group of six animals received a total light dose of 150 J $cm^{-2}$ (100 mW $cm^{-2}$). A second group of six animals received a total light dose of 450 J $cm^{-2}$ at the same dose rate. Vascular damage resulting from treatment was observed only at the final stages of the irradiation, despite the relatively high levels of fluorescence in the circulation. Immediate post-treatment (re)transplantation of the content of the chamber into the flank always resulted in tumour regrowth, confirming the presence of viable tumour cells following photodynamic therapy (PDT). When the chamber was left intact, the light dose of 450 J $cm^{-2}$ yielded complete tissue necrosis. The role of the dye–carrier complex in shielding the vascular surrounding from photoproducts was studied in a third group of animals. The presence of peroxides was demonstrated in the serum of these animals after PDT with zinc phthalocyanine in liposomes (ZnPc-lip) using a total light dose of 450 J $cm^{-2}$. This *ex vivo* observation supports the previously reported observations *in vitro* that the carrier complex is able to quench the photoproducts resulting from photoactivation of the photosensitiser which is present in the circulation.

In photodynamic therapy (PDT) the goal is to achieve tumour necrosis with minimal damage to normal tissue using local activation by light of a photosensitiser that is retained in the target tissue. PDT is currently undergoing clinical evaluation with Photofrin as photosensitiser, which is a preparation obtained from haematoporphyrin derivative (HPD) and enriched in the active fraction.

Phthalocyanines are being investigated as alternative photosensitisers for PDT (Ben-Hur & Rosenthal, 1986). They are structurally similar to porphyrins but in contrast have a high extinction coefficient in the 670–690 nm range. At this wavelength, tissue penetration of light is deeper than at 630 nm where porphyrins are excited.

Phthalocyanines have a flat aromatic macrocycle, which makes these compounds poorly water soluble. Adding sulphonic acid groups to the molecule (Brasseur *et al.*, 1987) yields photodynamically active, water-soluble derivatives. Their synthesis is relatively easy, but the subsequent purification can be quite elaborate. Cutaneous photosensitivity resulting from phthalocyanine administration is reported to be much less than from Photofrin (Tralau *et al.*, 1989). The dye is stable in a biological environment and shows fluorescence in its monomeric, active form. Its fluorescence can therefore be exploited for tumour-localising purposes but, in contrast to Photofrin, it also gives information on local dye concentration (MacRobert *et al.*, 1989). Phthalocyanines have been shown to photosensitise malignant cells both *in vitro* and *in vivo* [for a recent review see Rosenthal (1991) and van Lier (1990)].

Tumour localisation of the sulphonated phthalocyanines is greatly influenced by the lipophilic nature of the drug. The work of others (Chan *et al.*, 1990) and also our own observations have shown that fluorescence-based tumour localisation of sulphonated phthalocyanines is positively correlated with the degree of sulphonation of the phthalocyanine molecule (van Leengoed *et al.*, 1993*a*). However, where photodynamic activity with respect to tumour necrosis is concerned, the degree of sulphonation is inversely related to the effect, favouring lipophilic dyes over hydrophilic dyes (Brasseur *et al.*, 1988; Berg *et al.*, 1989; van Leengoed *et al.*, 1993*b*). Instead of adding sulphonate groups to the molecule to increase the water solubility, liposomes can be used as a carrier system, making the lipophilic drugs suitable for systemic administration. *In vivo*, liposomes associate with the lipid core of lipoproteins. Low-density lipoproteins (LDLs) have a particularly high affinity for tumour cells through a receptor-mediated transport mechanism (Goldstein *et al.*, 1979). Liposomes have been shown to facilitate efficient targeting *in vivo* by water-insoluble porphyrins (Jori *et al.*, 1983) as well as by Zn(II) phthalocyanine (ZnPc) (Reddi *et al.*, 1987) at very low dosages.

In this paper, we describe the *in vivo* fluorescence kinetics of this drug–carrier complex and determine the *in vivo* effects of subsequent optical irradiation on tumour necrosis using a dorsal skinfold chamber model. The role of the vascular effects resulting from treatment and quenching of photoproducts by the carrier complex of the photosensitiser present in the circulation will be discussed.

### Materials and methods

### *Animal model*

Female WAG/Rij rats (ITRI-TNO, Rijswijk, The Netherlands) 12–14 weeks of age were used. During a 3 week preparation period the animals were equipped with a skinfold chamber on their backs. The chamber includes a 0.5 mm layer of subcutaneous tissue, wedged between two transparent covers. An isogeneic mammary carcinoma was transplanted into the subcutaneous tissue of the chamber. The animal model permits observation of the pharmacokinetics of photosensitisers based on fluorescence and the assessment of vascular effects

Correspondence: H.L.L.M. van Leengoed
Received 14 October 1993; and in revised form 17 December 1993

resulting from photodynamic therapy (Star *et al.*, 1986; van Leengoed *et al.*, 1990). Hypnorm (Janssen Pharmaceuticals, Beerse, Belgium) was used as a general anaesthetic during all procedures. In accordance with the Dutch law on animal experiments, the protocol was submitted to and approved by the animal experiments committee.

*Drug-carrier system*

Zn(II) phthalocyanine (Ciba-Geigy, Basle, Switzerland) incorporated into small unilamellar liposomes was prepared as previously described (Valduga *et al.*, 1987). Briefly, 0.75-1 ml of a mixture of 66 μM ZnPc in pyridine (Janssen Pharmaceuticals) and 3.56 mM dipalmitoylphosphatidylcholine (DPPC) in ethanol was slowly injected into 0.9% aqueous sodium chloride at 55°C using a microsyringe. After spontaneous cooling to room temperature, the suspension was dialysed for 3 h against 2 × 500 ml of 0.9% saline to remove organic solvents. The resulting dye concentration was measured by optical absorbance using a Lambda 5 (Perkin Elmer) spectrophotometer ($\varepsilon = 2.42 \times 10^5\ M^{-1}\,cm^{-1}$ at $\lambda_{max}$). A calculated volume of the liposome suspension supplemented with phosphate-buffered saline (PBS) was administered via a tail vein of the animals resulting in a dye dosage of 0.14 mg per kg body weight.

*Fluorescence detection*

Fluorescence was excited at 610 nm with a fluence rate of 0.25 mW $cm^{-2}$ and detected above 665 nm using an RG665 coloured glass filter and an intensified CCD camera. Digitised fluorescence images obtained from the fluorescence detection system were used to measure average greyscale values of selected areas of interest of blood vessel, tumour and normal tissue. To minimise individual differences these values were used to express fluorescence of tumour tissue and blood vessels as averaged ratios relative to normal tissue (subcutis) values.

*Experimental procedure*

In order to establish the optimum dose for fluorescence detection and PDT, the first series of experiments was performed in a pilot study on 14 animals, varying drug dose, light dose and interval between drug administration and treatment. All experiments began when the tumours in the chambers had reached a diameter of 2-3 mm. Two drug doses were compared: 0.14 mg $kg^{-1}$ and 0.21 mg $kg^{-1}$. The animals were anaesthetised and, following the recording of an autofluorescence image, the dye-carrier complex was administered via a tail vein of the animal. At 5 min and at 24 h p.i., fluorescence images were recorded. Twenty-four hours after dye administration, a treatment light dose of 450 or 900 J $cm^{-2}$ was delivered to the chamber, using an irradiance of 100 mW $cm^{-2}$ at 610 nm, obtained from an argon ion pumped-dye laser system (Spectra Physics 171 + 375). The wavelength of 610 nm is at the fluorescence excitation maximum closest to the fluorescence emission wavelength of 675 nm (Figure 1). In this pilot study rhodamine was used as the lasing dye because fluorescence was the primary objective. With rhodamine it is not possible to generate light of 675 nm, the wavelength at which ZnPc has maximum light absorption. Therefore, when it was later decided to include PDT, this was performed at 610 nm. In the subsequent series of experiments DCM (4-dicyanomethylene-2-methyl-6(*P*-dimethylaminostytyl)-4H-pyran) was used as the lasing dye, because it allows generation of both 610 and 675 nm light (see below).

After treatment, the vascular effects were scored for a period of 7 days using a microscope at low magnification. After the observation period the content of the chamber was transplanted subcutaneously into the flank of the same animal to obtain an estimate of tumour cell viability.

All subsequent series of experiments were performed using a drug dose of 0.14 mg $kg^{-1}$ (see Discussion). In a group of six animals, fluorescence pharmacokinetics was observed at 5, 15, 30, 60, 120, 180, 240, 300 and 360 min and at 24 h p.i. Each digitised image was accompanied by a similar image of a piece of reference material (coloured plastic). This enabled correction of the fluorescence image for inhomogeneities in the detection system optics and variations in laser output. Twenty-four hours p.i. a treatment light dose of 150 J $cm^{-2}$ (100 mW $cm^{-2}$, 675 nm) was delivered to the chamber. During and after treatment, vascular effects of tumour and normal tissue were scored on a 0-8 scale. A score of 0 corresponds to no observable effect on the vasculature, whereas a score of 8 indicates no circulation. The phenomena associated with each score have been described in a previous paper (Van Leengoed *et al.*, 1993*b*). Two hours after treatment one chamber was prepared for histology and from two animals the content of the chamber was transplanted into the flank of the same animal. In the remaining three animals transplantation was performed at the end of a 7 day follow-up period during which the vascular damage was assessed daily. In order to achieve tumour necrosis, in a third group of six animals, the therapeutic light dose was increased to 450 J $cm^{-2}$.

The role of the dye-carrier complex in shielding the vascular surroundings from photodamage was studied in the serum of a fourth group of animals. The skinfold chambers of a group of six animals having received an i.v. dose of 0.14 mg $kg^{-1}$ ZnPc were irradiated 24 h p.i. with a total light dose of 450 J $cm^{-2}$. Immediately after treatment the serum was collected and stored frozen at − 20°C until it could be tested for the presence of any products due to photosensitisation by the phthalocyanine (still detectable by means of fluorescence in the circulation). Special attention was paid to serum lipoproteins, which are the specific carriers of liposome-delivered ZnPc (Reddi *et al.*, 1990). The lipoprotein fraction of serum proteins was separated by density-gradient ultracentrifugation (Havel *et al.*, 1955). The lipoproteins were resuspended in saline and divided into two aliquots. One aliquot was used for determining the apoprotein aromatic amino acids after denaturation obtained by dilution with an excess of 2% aqueous sodium dodecyl sulphate (SDS); the measurements were performed by a MPF-4 Perkin Elmer spectrophotofluorimeter, using an excitation wavelength of 285 nm. The second aliquot was used for evaluation of lipid peroxidation in terms of malondialdehyde (MDA) formation; this was based on the thiobarbituric (TBA) assay (Kappus, 1985) which allows quantitative measure of some decomposition products of lipid peroxides. The MDA concentration was determined by absorbance measurements at 535 nm using a molar extinction coefficient of 154,000 $M^{-1}\,cm^{-1}$. Lipoprotein samples from animals that had been treated by the same procedure but that had not been exposed to light were analysed in parallel and used as controls.

**Figure 1** Absorption spectrum of zinc phthalocyanine in pyridine (ZnPc concentration 0.0074 mg $ml^{-1}$). The two excitation wavelengths used in this study, 610 and 675 nm, are indicated by arrows.

**Table I** Results of the pilot experiment performed to determine the drug and light dose and treatment interval to be used in the main study. Treatment light of 610 nm wavelength was delivered to the observation chamber at an irradiance of $100\,mW\,cm^{-2}$. The reason for the choice of this wavelength is explained in the text

| *ZnPc dose ($mg\,kg^{-1}$)* | *Number of animals with tumour fluorescence > subcutis at 24 h p.i.* | *PDT light dose ($J\,cm^{-2}$)* | *Complete necrosis of tissue in chamber for the given interval to treatment* | | *Regrowth after retransplantation of tumour into flank (no. of tumours)* |
|---|---|---|---|---|---|
| | | | *24 h* | *48 h* | |
| 0.14 | 4/4 | 450 | 0/2 | –/– | 2/2 |
| | | 900 | 1/2 | –/– | 1/2 |
| 0.21 | 3/10 | 450 | 1/2 | 1/2 | 2/4 |
| | | 900 | 1/4 | 1/2 | 4/6 |

### Results

In the pilot experiments, both fluorescence excitation and photodynamic therapy were performed using light of 610 nm (see Materials and methods). The results of these experiments are summarized in Table I.

Tumour fluorescence showed little selectivity with $0.21\,mg\,kg^{-1}$ ZnPc compared with $0.14\,mg\,kg^{-1}$ and exceeded the background in only 3 out of 10 animals. Complete necrosis was observed at both light dosages using a drug dose of $0.21\,mg\,kg^{-1}$, but a light dose of $900\,J\,cm^{-2}$ was required when a drug dose of $0.14\,mg\,kg^{-1}$ was used. When retransplantation was done immediately following therapy, tumour regrowth was observed with both drug doses.

Figure 2 shows digital fluorescence images of liposome-bound phthalocyanine in the chamber model before and at four different intervals after i.v. administration of $0.14\,mg\,kg^{-1}$. Note that the fluorescence of tumour and blood vessel increases following administration and that even at 24 h both tumour and blood vessel fluorescence exceed that of the normal tissue.

The fluoresence signals of tumour, blood vessel and normal tissue following i.v. administration of the drug are expressed as averaged greyscale values in Figure 3. After the dye has been administered, fluorescence in the blood vessels increases slowly, reaching a plateau at 120 min p.i. The fluorescence signal then remains stable but the ratio of vessel to subcutis starts to decline (Figure 4) because of the slow increase of fluorescence in the normal tissue. Tumour tissue *vs* normal tissue ratios increase even more slowly, reaching a maximum value approximately 4 h p.i. Two hours p.i. a ratio of 2 is exceeded, and this level is maintained at least over a 4 h period between 2 and 6 h p.i. From 60 min onwards and at 24 h p.i., the fluorescence in both the tumour and the circulation exceeds that of the normal tissue.

Twenty-four hours p.i., a treatment light dose of $150\,J\,cm^{-2}$ ($100\,mW\,cm^{-2}$, 675 nm) was administered to the chamber. This light dose was based on a ratio of about 6 between the optical absorption of ZnPc at 675 and 610 nm (see also Discussion). With this light dose complete necrosis was never observed. Retransplantation, either 2 h after treatment or at the end of the observation period, consistently resulted in tumour regrowth. The presence of vital tumour cells was confirmed by histology.

In order to achieve tumour necrosis it was decided to increase the dose to $450\,J\,cm^{-2}$ and the averaged circulation damage scores of the third group of six animals are shown in Figure 5. Using this light dose, maximum damage scores were observed in tumour tissue starting at day 1. A small

**Figure 2** Digitised fluorescence images of ZnPc-lip taken before injection (centre) and at 5 (**a**), 60 (**b**) and 360 min (**c**) and at 24 h p.i. (**d**). Note the tumour fluorescence at 360 min and the persistence of fluorescence in the circulation. In terms of average greyscale values, fluorescence of the tumour still exceeds values of the normal tissues at 24 h p.i. The diameter of visible tissue in the chamber is 9 mm.

**Figure 3** Fluorescence pharmacokinetics of ZnPc-lip following i.v. administration of $0.14\,mg\,kg^{-1}$ of the drug–carrier complex. Fluorescence of the regions of interest (■, tumour; □, vessel; and (▨, normal tissue) was measured in the digitised image for each of the six animals and at each time point and expressed as greyscale values. Error bars indicate s.e.m.

**Figure 4** Averaged ratios ± s.e.m. of tumour tissue (■) and blood vessel (□) fluorescence relative to normal tissue (subcutis) fluorescence following dye administration ($0.14\,mg\,kg^{-1}$).

**Figure 5** Circulation damage scores of tumour (■) tissue and normal (□) tissue, during and after a treatment light dose of 450 J $cm^{-2}$ (100 mW $cm^{-2}$, 675 nm) 24 h after i.v. administration of ZnPc-lip. Scores range from 0 (no observable damage) to 8 (no observable circulation). Values are averaged over six animals and the duration of the treatment is expressed in minutes. Zero days post treatment indicates 2 h after the end of the irradiation, which lasted 75 min.

recovery of the circulation in the normal tissue of one animal resulted in a drop in the average damage score to below 8, but the damage score in tumour tissue remained maximal during the observation period. Complete necrosis without recovery was observed in all three rats that were monitored during the whole observation period. When the content of the chamber was retransplanted 2 h after treatment, regrowth was observed. Histology performed at the same interval again demonstrated the presence of vital tumour cells. During treatment the vascular effects appeared minimal, increasing only during the last 15 min of the total treatment time of 75 min.

The lipoproteins isolated from the serum of a fourth group of six animals which received ZnPc PDT with 450 J $cm^{-2}$ were analysed as described in the Materials and methods section. The 285 nm excited apoprotein fluorescence gave a maximum at 358 nm, which is typical of tryptophyl residues in denatured protein samples; the spectral shape and relative fluorescence intensity were essentially identical for lipoproteins obtained from irradiated and unirradiated serum. This observation strongly suggests that tryptophan, a main target of porphyrin-photosensitised protein damage (Jori & Spikes, 1984), is not affected under our irradiation conditions. On the other hand, the analysis for lipid peroxidation showed the formation of 3.97 μmol of MDA per lipoprotein molecule *vs* a background value of 0.18 μmol of MDA found for unirradiated serum lipoprotein.

### Discussion

Hydrophobic photosensitisers like ZnPc, which have excellent photochemical and photophysical properties for PDT, but will aggregate and/or precipitate in an aqueous environment, can be incorporated into liposomes and administered systemically. In this way about 30% of lipsome-bound photosensitisers can be selectively transferred to LDLs (Jori, 1989). These lipoproteins can be endocytosed by neoplastic cells through a specific receptor-mediated pathway. Several malignant tissues have elevated numbers of LDL receptors (Spikes & Jori, 1987). A large amount of drug can thus be accumulated by the tumour tissue in spite of the low injected doses (Reddi *et al.*, 1987).

During the pilot experiment, laser light of 675 nm was not available. The photosensitiser was therefore excited at 610 nm, a local maximum in the absorption spectrum (Figure 1). This wavelength was also used for fluorescence excitation. Based on the results of this pilot study a sensitiser dose of 0.14 mg $kg^{-1}$ was selected as optimal to study both fluorescence pharmacokinetics and photodynamic effects of the drug–carrier complex. Although a larger photosensitiser dose was expected to result in increased phototoxicity, the increase in normal tissue fluoresence using 0.21 mg $kg^{-1}$ had an adverse effect on the tumour to normal tissue fluorescence ratio. The sensitiser dose of 0.14 mg $kg^{-1}$ in combination with treatment light of 610 nm and a total light dose of 900 J $cm^{-2}$ was sufficient to cause tumour necrosis. This light dose was rather high but should be viewed with regard to the low extinction coefficient of ZnPc at this wavelength (see below for 675 nm).

Phthalocyanine fluorescence can be employed as a diagnostic tool to monitor the presence of the photosensitiser in its monomeric, active state (MacRobert *et al.*, 1989). Detecting HPD or Photofrin in this way is more complicated because these drugs contain a number of components with different relationships between fluorescence and photodynamic activity.

After administration of the dye–liposome complex, a gradual accumulation of fluorescence in the circulation as well as in the tumour (Figure 3) is observed. When studying sulphonated phthalocyanines in the same chamber model, a fluorescent angiogram with the highest intensity in this circulation is usually observed immediately p.i., followed by a gradual decrease of the fluorescence in time (van Leengoed *et al.*, 1990). However, the fluorescence in the circulation reached its peak only 120 min p.i. using liposomal ZnPc. This increase in the fluorescence in the circulation till 120 min p.i. might be explained by considering the self-quenching of fluorescence owing to the relatively high concentration of the photosensitiser in the carrier system. This increase in fluorescence could represent the release process by the carrier system of the photosensitiser similar to that which has been described for fluorescein (Weinstein *et al.*, 1977).

The fluorescence signal in tumour tissue increases even more slowly than the fluorescence in the circulation. This observation probably relates to the fact that the liposome-bound ZnPc is taken up by lipoproteins before being accumulated by the tumour cells through an active process. Between 3 and 5 h p.i. the tumour fluorescence exceeds a ratio of 2 (Figure 4), making it easily detectable against the normal tissue fluoresence, which hardly increases during this interval. This interval could therefore be utilised as a window for tumour detection. The limited increase in fluorescence in the normal tissue also contributes to the fact that at 24 h p.i. both tumour and blood vessel fluorescence remains detectable. When tested in the same tumour model system at 24 h p.i., HPD and Photofrin fluorescence usually appeared lower in the tumour than in the surrounding normal tissue (Van Leengoed *et al.*, 1990).

Exciting the photosensitiser at 610 nm with 900 J $cm^{-2}$ resulted in tumour necrosis in one out of two animals using a sensitiser dose of 0.14 mg $kg^{-1}$. When the sensitiser was excited at 675 nm, a 6-fold reduction in the total light dose to 150 J $cm^{-2}$ was expected to yield similar results. This idea was based on the fact that, in pyridine, phthalocyanine absorption at 675 nm appeared at least 6-fold larger than at 620 nm (Figure 1). Actually, using this light dose, 1 day after treatment the vasculature of the normal tissue was already recovering and tumour regrowth was always observed. Tumour necrosis was achieved when a light dose of 450 J $cm^{-2}$ was used, but this light dose also caused a maximal effect on the normal tissue circulation. To explain these observations we propose that *in vivo* aggregation of the dye may occur, and this will result in a reduction of the differences in optical absorption as a function of wavelength.

A drug dose of 0.14 mg $kg^{-1}$ can be considered very low compared with the dose of Photofrin (10 mg $kg^{-1}$) or sulphonated phthalocyanines (2.5–5 mg $kg^{-1}$) used in the experiments with rats. The fact that tumour necrosis was observed with this low drug dose suggests that the targeting of the photosensitiser using liposomes as carriers is effective.

Targeting of the photosensitiser towards individual tumour cells is expected to result in more direct cell kill (Zhou *et al.*, 1988). However, from our data it appears that the number of tumour cells killed directly is not sufficient for tumour control. Retransplantation immediately after treatment always resulted in tumour regrowth. Moreover, viable tumour cells were confirmed histologically 2 h after treatment. Tumour cell kill cannot be quantified using our method. It only allows the demonstration of the presence or absence of tumour cells capable of causing tumour regrowth. Tumour control by PDT with ZnPc-lip therefore probably requires vascular damage in a margin of normal tissue around the tumour.

During the first 5 min of illumination, vasospasm of arteries was the most striking vascular effect that was observed. This vasospasm usually disappeared within the first 30 min of irradiation. In comparable experiments using sulphonated phthalocyanines similar vasospasm of the arteries was observed. These effects were stronger using dyes chelated with zinc as a central metal ion rather than with aluminium. The vasospasms were considered to cause hypoxia and, assuming that oxygen is indispensable for Pc-PDT (Bown *et al.*, 1986), thereby negatively influence the effectiveness of the photochemical reaction. Using liposome-bound ZnPc it seems that after the initial response of the artery to the treatment arterial blood supply is restored till the end of treatment. During the period of illumination no thrombi or vasoconstriction of the larger veins could be observed. By the end of the treatment period stasis was observed predominantly in the capillary bed of both tumour and surrounding normal tissue. However, judging from the initial vasospasms and damage to the capillary bed, the endothelium is likely to be a target for PDT using ZnPc-lip. These findings agree with what has been found by Milanesi *et al.* (1987) and Evensen (1983). The vascular effects reach their maximum values between 2 h post therapy and day 1 (Figure 4) and cannot be distinguished from the inflammatory response to the treatment.

At day 1, fluorescence in the blood vessels was clearly distinguishable from the normal tissue, so that active photosensitisers appear to be present in the circulation. In view of this observation, the vascular effects observed were much less than expected. This could well be a consequence of a protective action performed by the lipoprotein carrier of ZnPc. The close spatial interrelation between the photosensitiser and unsaturated lipids in the large lipid moiety of lipoproteins should favour a preferential attack of the photogenerated reactive transients (e.g. singlet oxygen or radical species on intramolecular targets, thus reducing the concentration of toxic intermediates available for attacking the vessel wall. In other words, lipoprotein carriers might act as a quencher in the photochemical process, as has been shown earlier *in vitro* for porphyrin photosensitisation of LDLs. (Candide *et al.*, 1988; Maziere *et al.*, 1990).

The study presented in this paper describes the fluorescence pharmacodynamics and vascular damage following photodynamic therapy using phthalocyanine administered in liposomes. Because of this combination we were not able to study the fluorescence kinetics beyond 24 h p.i. and therefore do not know how quickly the dye is eliminated from the tumour tissue. By combing the study of fluorescence and PDT we meant to obtain as much information as possible with a limited number of experimental animals, considering that the model is quite laborious. We chose to treat at the rather common interval of 24 h p.i. Based on the information available now and described in this paper it should be worthwhile to set up new experiments to study the fluorescence kinetics over longer time periods after drug administration and also to study the effects of PDT performed at the time of the highest fluorescence ratio between tumour and normal tissues.

This work was supported by the Dutch Cancer Society ('Koningin Wilhelmina Fonds), Project DDHK-89-3. Funds for equipment were granted by the 'Maurits and Anna de Kock Stichting', the 'Nijbakker Morra Stichting' and the 'Josephine Nefkens Stichting'. The authors wish to thank Dennes van der Wel for preparing the photographs.

Abbreviations: HPD, haematoporphyrin derivative; i.v. intravenous; LDL, low-density lipoprotein; MDA, malondialdehyde; PDT, photodynamic therapy; p.i., post injection; ZnPc-lip; zinc phthalocyanine in liposomes.

### References

BEN-HUR, E. & ROSENTHAL, I. (1986). Action spectrum (600-700 nm) for chloraluminum phthalocyanine induced photoxicity in chinese hamster cells. *Lasers Life Sci.*, **1**, 79-86.

BERG, K., BOMMER, J.C. & MOAN, J. (1989). Evaluation of sulphonated aluminium phthalocyanines for use in photochemotherapy. Cellular uptake studies. *Cancer Lett.*, **44**, 7-15.

BOWN, S.G., TRALAU, C.J., COLERIDGE-SMITH, P.D., AKDEMIR, D. & WIEMAN, T.J. (1986). Photodynamic therapy with porphyrin and phthalocyanine sensitization: quantitative studies in normal rat liver. *Br. J. Cancer*, **54**, 43-52.

BRASSEUR, N., ALI, H., LANGLOIS, R., WAGNER, R., ROUSSEAU, J. & VAN LIER, J.E. (1987). Biological activities of phthalocyanines. V. Photodynamic therapy of EMT-6 mammary tumors in mice with sulphonated phthalocyanines. *Photochem. Photobiol.*, **45**, 581-586.

BRASSEUR, N., ALI, H., LANGLOIS, R. & VAN LIER, J.E. (1988). Biological activities of phthalocyanines. IX. Photosensitization of V-79 chinese hamster calls and EMT-6 mouse mammary tumor by selectively sulphonated zinc phthalocyanines. *Photochem. Photobiol.*, **47**, 705-711.

CANDIDE, C., REYFTMANN, J., SANTUS, R., MAZIERE, J.C., MORLIERE, P. & GOLDSTEIN, S. (1988). Modification of epsilon-amino groups of lysines, cholesterol oxidation and oxidized lipid-apoprotein cross-link formation by porphyrin-photosensitized oxidation of human low density lipoproteins. *Photochem. Photobiol.*, **48**, 137-146.

CHAN, W., MARSHALL, J., SVENSEN, R., BEDWELL, J. & HART, I.R. (1990). Effect of sulphonation on the cell and tissue distribution of the photosensitiser aluminium phthalocynaine. *Cancer Res.*, **50**, 4533-4538.

EVENSEN, J.F., GALDAL, K.K. & NILSEN, E. (1983). LDL-induced cytotoxicity and its inhibition by anti-oxidant treatment in cultured endothelial cells and fibroblasts. *Atheroschlerosis*, **49**, 23-30.

GOLDSTEIN, J.L., ANDERSON, R.G.W. & BROWN, M.S. (1979). Coated pits, coated vesicles and receptor-mediated endocytosis. *Nature*, **279**, 679-685.

HAVEL, R.J., EDER, H.A. & BRAGDON, J.H. (1955). Distribution and chemical composition of ultracentrifugally separated lipoproteins in human serum. *J. Clin. Invest.*, **34**, 1345-1353.

JORI, G., TOMIO, L., REDDI, E., ROSSI, E., CORTI, L., ZORAT, P.L. & CALZAVARA, F. (1983). Preferential delivery of liposome-incorporated porphyrins to neoplastic cells in tumor-bearing rats. *Br. J. Cancer*, **48**, 307-309.

JORI, G. & SPIKES, J.D. (1984). Photochemistry of porphyrins. In *Topics in Photomedicine*, Smith, K. (ed.) pp. 193-212. Plenum Press: New York.

JORI, G. (1989). In vivo transport and pharmacokinetic behaviour of tumour photosensitizers. In *Photosensitizing Compounds: their Chemistry, Biology and Clinical Use*. Bock, G. & Harnett, S. (eds.) pp. 78-86. (Ciba Foundation Symposium 146). John Wiley: Chichester.

KAPPUS, H. (1985). Lipid peroxidation: mechanisms analysis, enzymology and biological relevance. In *Oxidative Stress*, Sies, H. (ed.) pp. 273-310. Academic Press: New York.

MACROBERT, A.J., BOWN, S.G. & PHILLIPS, D. (1989). What are the ideal photoproperties for a sensitizer? In *Photosensitizing Compounds: their Chemistry, Biology and Clinical Use*, Bock, G. & Harnett, S. (eds.) pp. 4-16. (Ciba Foundation Symposium 146). John Wiley: Chichester.

MAZIERE, J.C., SANTUS, R., MORLIERE, P., REYFTMANN, J., CANDIDE, C., MORA, L., SALMON, S., MAZIERE, C., GATT, S. & DUBERTRET, L. (1990). Cellular uptake and photosensitizing properties of anticancer porphyrins in cell membranes and low and higher density lipoproteins. *J. Photochem. Photobiol. B: Biology*, **6**, 61-68.

MILANESI, C., BIOLO, R., REDDI, E. & JORI, G. (1987). Ultrastructural studies on the mechanism of the photodynamic therapy of tumors. *Photochem. Photobiol.*, **46**, 675–682.

REDDI, E., LO CASTRO, G., BIOLO, R. & JORI, G. (1987). Pharmacokinetics studies with zinc(II)phthalocyanine in tumour bearing mice. *Br. J. Cancer*, **56**, 597–600.

REDDI, E., ZHOU, C., BIOLO, R., MENEGALDO, E. & JORI, G. (1990). Liposome or LDL administered Zn(II)phthalocyanine as a photodynamic agent for tumours. I. Pharmacokinetic properties and therapeutic efficiency. *Br. J. Cancer*, **61**, 407–411.

ROSENTHAL, I. (1991). Phthalocyanines as photodynamic sensitizers. *Photochem. Photobiol.*, **53**, 859–870.

SPIKES, J.D. & JORI, G. (1987). Photodynamic therapy of tumours and other diseases. *Lasers Med. Sci.*, **2**, 3–15.

STAR, W.M., MARIJNISSEN, J.P.A., VAN DEN BERG-BLOK, A.E., VERSTEEG, A.A.C., FRANKEN, N.A.P. & REINHOLD, H.S. (1986). Destruction of rat mammary tumor and normal tissue microcirculation by hematoporphyrin derivative photoradiation observed in vivo in sandwich observation chambers. *Cancer Res.*, **46**, 2532–2540.

TRALAU, C.J., YOUNG, A.R., WALKER, N.P.J., VERNON, D.I., MACROBERT, A.J., BROWN, S.B. & BOWN, S.G. (1989). Mouse skin photosensitivity with dihaematoporphyrin ether (DHE) and aluminium sulphonated phthalocyanine (AlSPc): a comparative study. *Photochem. Photobiol.*, **49**, 305–312.

VALDUGA, G., REDDI, E. & JORI, G. (1987). Spectroscopic studies on Zn(II)-phthalocyanine in homogeneous and microheterogeneous systems. *J. Inorg. Biochem.*, **29**, 59–65.

VAN LEENGOED, E., VERSTEEG, A.A.C., VAN DER VEEN, N., VAN DEN BERG-BLOK, A.E., MARIJNISSEN, J.P.A. & STAR, W. (1990). Tissue-localizing properties of some photosensitizers studied by in vivo fluorescence imaging. *J. Photochem. Photobiol. B: Biology*, **6**, 111–119.

VAN LEENGOED, H.L.L.M., VAN DER VEEN, N., VERSTEEG, A.A.C., OUELLET, R., STAR, W. & VAN LIER, J.E. (1993*a*). In vivo fluorescence kinetics of phthalocyanines in a skin-fold observation chamber model: role of central metal ion and degree of sulfonation. *Photochem. Photobiol.*, **58**, 233–237.

VAN LEENGOED, H.L.L.M., VAN DER VEEN, N., VERSTEEG, A.A.C., OUELLET, R., VAN LIER, J.E. & STAR, W.M. (1993*b*). In vivo photodynamic effects of phthalocyanines in a skin-fold observation chamber model: Role of central metal ion and degree of sulphonation. *Photochem. Photobiol.*, **58**, 575–580.

VAN LIER, J.E. (1990). Phthalocyanines as sensitizers for PDT of cancer. In *Photodynamic Therapy of Neoplastic Disease*. Vol. 1, Kessel, D. (ed.) pp. 279–290. CRC Press: Boston.

WEINSTEIN, J.N., YOSHIKAMI, S., HENKART, P., BLUMENTHAL, R. & HAGINS, W.A. (1977). Liposome cell interaction: transfer and intracellular release of a trapped fluorescent marker. *Science*, **195**, 489–491.

ZHOU, C., MILANESI, C. & JORI, G. (1988). An ultrastructural comparative evaluation of tumors photosensitized by porphyrins administered in aqueous solution, bound to liposomes or to lipoproteins. *Photochem. Photobiol.*, **48**, 487–492.